# A Case of Syphilis by Conception

**Published:** 1894-02

**Authors:** 

**Affiliations:** Bordeaux, France


### A CASE OF SYPHILIS BY CONCEPTION.*

By PROF. CHAMBRELENT,

BORDEAUX, FRANCE.

A woman, twenty-six years of age, who was delivered in the service of the obstetric clinic, June 6th, 1893, forms the subject of this article. Nothing very particular about her hereditary antecedents. Nothing in her personal antecedents up to the age of twenty, that is, up to 1887, the period in which an important factor entered the life of this woman—her husband.

Her husband is a man now aged thirty-four, of vigorous constitution, who did not present anything particular until 1884, the last

*Translated from the Archives de Tocologie et de Gynecologie, by J. McF. Gaston, Jr., A.M., M.D., Atlanta, Ga.

year of his military service. This year he contracted syphilis, the primary and secondary stages of which were very well recognized and treated consequently by mercury, and healed perfectly. At the time of his marriage no symptoms were visible. He even consulted a physician who allowed him to marry. His wife became pregnant almost immediately, and at four months aborted without presenting any other sign, either primary or secondary.

Almost immediately afterwards a second pregnancy occurred. This woman then presented some symptoms about the nature of which there is no possible doubt: night headache, sore throat, eruptions on her skin, etc. But, as a fact of the highest importance, no initial lesion appeared and absolutely nothing on the part of the genital organs. This woman formally denies it, and what confirms her statement, is that there has not been any enlargement of the lymphatic glands in the groin. Her second pregnancy terminated like the first, by an abortion at four months.

The following year a third pregnancy occurred. This went to full term, but the child was born dead and macerated.

In 1891, she became pregnant for the fourth time, which also went to term, and this time the child was born alive, but died on the following day.

This was then her fifth pregnancy. Everything passed normally, the woman was delivered at term of a child weighing 3,050 grammes and seemed to have a good constitution. A slight elevation of temperature which came on June 8th, necessitated the moving of the patient, and she was placed in my clinic.

The child had decreased in weight very much, much more than it is usual for the new-born during the two or three days following birth. On the fourth day, the examination of the hands and feet of this child displayed reddened spots, well defined, well circumscribed, which soon passed into a condition of vesicles, no doubt being the vesicular eruption of pemphigus. The intensive mercurial treatment was immediately instituted and a notable improvement was produced. The child increased in weight during four or five days, but began again to decrease in weight afterwards, and presented the gravest general symptoms, which caused us to give an extremely reserved prognosis.

There was evidently in the history of this woman withal something rare, for it departs from the usual evolution of syphilis which is pointed out in analogous cases. The woman had never, indeed, presented the primary symptoms; she had never had chancre, neither on her genital organs nor any other part of her body. Doubtless this chancre may have passed by unperceived as it frequently does, but we repeat that there is no enlargement of the lymphatic glands, and there never has been any.

We are then in the presence of that particular form of syphilis, a species of aborted syphilis, according to the happy expression of Professor Fournier, to which the name of syphilis by conception has been given.

This is what takes place in these cases: A syphilitic individual has intercourse with his wife and does not contaminate her. As long as this woman does not conceive, there is no sympton. She becomes pregnant, and immediately the secondary symptoms of syphilis appear. This syphilis well deserves the name of syphilis by conception, because it was the fetus which had rendered its mother syphilitic. And, in general, among the secondary symptoms, that which predominates is headache, well defined at night, which is often most intense, and against which it is necessary to be on guard. If, indeed, it is not attributed to its true cause, as has often happened, of course the physician is surprised to find himself powerless against it, to see it resist the most rigorous treatment. I wish to cite a case of this particular nature, a woman who is now in the service of Professor Pitres. This patient suffered horribly with a headache which was proof against all the means of treatment tried up to that time. As she had just aborted, I suspected the true cause of this headache, and indeed, as soon as the specific treatment was instituted, a very notable improvement was seen.

This syphilis by conception is not rare; it has been especially studied by two French syphilographers, M. M. Diday, of Lyon, and Fournier. They have made observations absolutely well-defined, among which the following of M. Gailleton, quoted by Fournier, seems particularly conclusive.

A young woman, sixteen years of age, had a single intercourse with a young man who had been affected with syphilis for six months, treated regularly and freed of all signs for one month past. On the day following his only intercouse, he was examined with care by M. Gailleton who did not find any eruption on his body or any trace of syphilis. But this intercourse had impregnated the unfortunate girl; at the end of two months she complained of violent headache, then of other secondary symptoms of syphilis. Finally, at the end of nine months, she was delivered of a child, who, fifteen days after birth, presented indubitable signs of syphilis.

Even further, a woman may have absolutely no symptom and yet have been rendered syphilitic by the fetus. Then the syphilis is said to be latent. We see cases of this kind when a new–born syphilitic child, having visible signs of syphilis, is nursed by its mother, without the latter having the least specific symptom.

The following note of Neumann is absolutely conclusive:

A young girl, sound until she bore a syphilitic child, which she nursed, and which, although affected with numerous patches on the lips, did not communicate any disease to her. The grandmother of the child having one day kissed the child upon its lips, contracted a chancre of the lip, soon followed by secondary symptoms.

And if sometimes, a long time after, tertiary symptoms supervene, very often there is no symptom during the whole life of the woman thus attacked with latent syphilis.

It was impossible that this kind of vaccination of the mother against syphilis should not be verified by inoculation of the specific virus. But it was necessary, to attempt this experiment, to have a great boldness which was only to be obtained by an absolutely sure diagnosis. The experiments have, however, been performed twice, and the results have been such as could have been easily foreseen. Caspary was led to practice this inoculation under the following circumstances:

A man forty years old, married, having healthy children, contracted syphilis in 1872. His wife having been notified, avoided all intercourse until the period when her husband was considered inoffensive. She was then examined with the greatest care and has never presented the least symptoms. In October, 1872, she became pregnant and aborted at six months. Gummas were thought to be on the placenta. Wishing to see whether this woman was syphilitic or not before beginning a specific treatment with her, Dr. Caspary proposed to her to judge the question by inoculation of the virus. The woman accepted the proposition; pus from the mucous patch was taken from a syphilitic man who had not yet undergone any treatment. This pus was inoculated with a lancet into the arm of this woman. The inoculation was watched closely but did not give the least symptom.

[A case that matched that of Professor Chambrelent occurred in the practice of Dr. J. McFadden Gaston, during his residence in Brazil, South America. An American lady, aged nineteen, of good family, had married an Englishman, aged twenty-eight, who had had syphilis but thought himself well, and on his own responsibility stopped all treatment. Dr. Gaston had treated him for syphilis before his marriage, when he had ulcerated inguinal glands but was not consulted as to his marriage. He married clandestinely, contrary to the wishes of the young lady's family.

Before full term the woman gave birth to a dead fetus that was macerated with exfoliation of skin. Another birth occurred of a similar character. There were more than two births of dead feti, one fetus being of over six months, and the last of full term.

Finally, after a thorough treatment of both husband and wife, a healthy child was born, and gave no subsequent signs of syphilis.

The indications of secondary syphilis were unmistakable in the mother, however, up to the second birth. The mother of the syphilitic children had bullæ of the pemphigus variety, but the father showed no signs.

Immediate improvement followed the use of iodide of potassium and protiodide of mercury, both of which had been used after the birth of the first dead fetus. A persistent and increased use of the mixed treatment evidently effected a cure.—Translator.]

